# mRNA lipid nanoparticles expressing cell-surface cleavage independent HIV Env trimers elicit autologous tier-2 neutralizing antibodies

**DOI:** 10.3389/fimmu.2024.1426232

**Published:** 2024-07-25

**Authors:** Javier Guenaga, Mehrdad Alirezaei, Yu Feng, Mohamad-Gabriel Alameh, Wen-Hsin Lee, Sabyasachi Baboo, Jocelyn Cluff, Richard Wilson, Shridhar Bale, Gabriel Ozorowski, Paulo Lin, Ying Tam, Jolene K. Diedrich, John R. Yates, James C. Paulson, Andrew B. Ward, Drew Weissman, Richard T. Wyatt

**Affiliations:** ^1^ Wyatt Lab, Department of Immunology and Microbiology, Scripps Research, La Jolla, CA, United States; ^2^ Weissman Lab, Penn Institute for RNA Innovation, Perelman School of Medicine, University of Pennsylvania, Philadelphia, PA, United States; ^3^ Weissman Lab, Department of Pathology and Laboratory Medicine, Perelman School of Medicine, University of Pennsylvania, Philadelphia, PA, United States; ^4^ Ward Lab, Department of Integrative Structural and Computational Biology, Scripps Research, La Jolla, CA, United States; ^5^ Scripps Consortium for HIV/AIDS Vaccine Development (CHAVD), Scripps Research, La Jolla, CA, United States; ^6^ Acuitas Therapeutics, Vancouver, BC, Canada; ^7^ Paulson Lab, Department of Molecular Medicine, Scripps Research, La Jolla, CA, United States; ^8^ Weissman Lab, Department of Medicine, Perelman School of Medicine, University of Pennsylvania, Philadelphia, PA, United States

**Keywords:** HIV, vaccine, mRNA, NFL, SOSIP, Env, immunogenicity

## Abstract

The HIV-1 envelope glycoprotein (Env) is the sole neutralizing determinant on the surface of the virus. The Env gp120 and gp41 subunits mediate receptor binding and membrane fusion and are generated from the gp160 precursor by cellular furins. This cleavage event is required for viral entry. One approach to generate HIV-1 neutralizing antibodies following immunization is to express membrane-bound Env anchored on the cell-surface by genetic means using the natural HIV gp41 transmembrane (TM) spanning domain. To simplify the process of Env trimer membrane expression we sought to remove the need for Env precursor cleavage while maintaining native-like conformation following genetic expression. To accomplish these objectives, we selected our previously developed ‘native flexibly linked’ (NFL) stabilized soluble trimers that are both near-native in conformation and cleavage-independent. We genetically fused the NFL construct to the HIV TM domain by using a short linker or by restoring the native membrane external proximal region, absent in soluble trimers, to express the full HIV Env ectodomain on the plasma membrane. Both forms of cell-surface NFL trimers, without and with the MPER, displayed favorable antigenic profiles by flow cytometry when expressed from plasmid DNA or mRNA. These results were consistent with the presence of well-ordered cell surface native-like trimeric Env, a necessary requirement to generate neutralizing antibodies by vaccination. Inoculation of rabbits with mRNA lipid nanoparticles (LNP) expressing membrane-bound stabilized HIV Env NFL trimers generated tier 2 neutralizing antibody serum titers in immunized animals. Multiple inoculations of mRNA LNPs generated similar neutralizing antibody titers compared to immunizations of matched NFL soluble proteins in adjuvant. Given the recent success of mRNA vaccines to prevent severe COVID, these are important developments for genetic expression of native-like HIV Env trimers in animals and potentially in humans.

## Introduction

The HIV-1 envelope glycoprotein (Env) is the only virally encoded neutralizing determinant on the surface of the virus. The gp120 and gp41 Env subunits mediate receptor binding and membrane fusion, respectively. These subunits are generated from precursor cleavage of the trimeric gp160 by cellular furins, liberating the gp41 fusion peptide (FP), required for viral-to-cell membrane fusion and entry. Following furin-mediated activation, the entry-competent trimer of gp120 and gp41 heterodimers remain associated in a metastable non-covalent manner (1, 2). To elicit Env-directed neutralizing antibodies *in vivo* directed at the functional spike, investigators in the HIV vaccine field have developed spike mimetics as the first step toward a broadly effective vaccine. To a large extent, the generation of native-like HIV-1 Env spikes has focused on soluble trimers, led by the so-called Env mimics “SOSIP” (3, 4). The gp140 SOSIP trimers require the same furin-mediated cleavage for native-like conformation along with a helix destabilizing mutation in gp41, I559P (5, 6). An improvement of the SOSIP design is to remove the need for furin cleavage by introduction of a flexible linker between the gp120 and gp140 subunits to generate soluble “NFL” trimers (for native flexibly linked) (7, 8). Both SOSIP and NFLs can be produced as recombinant proteins and are being evaluated in the clinic as vaccine candidates. However, production of these recombinant trimers as GMP-grade clinical material is time consuming and expensive. In the likely event that prime:boosting of multiple HIV-1 Env components or strains is required to elicit cross-neutralizing antibodies, then genetic expression should reduce time and cost. The rapid design, development, and vaccination of humans as exemplified by mRNA vaccines expressing multiple variants of membrane-bound SARS-CoV2 spike proteins is strong evidence for such an approach.

The propensity of Env to generate well-ordered native-like trimeric proteins is crucial for the success of a nucleic acid HIV vaccine as genetic expression *in vivo* does not allow for the removal of undesired, and non-native forms of Env. These off-target conformers generate ‘immune distracting’ antibody responses against non-native and non-neutralizing determinants. Because the HIV-1 Env is normally expressed with transmembrane anchor points, soluble versions of Env, truncated following residue 664, lack the hydrophobic membrane proximal external region (MPER) and natural transmembrane anchor points and often require internal stabilizing mutations to generate mostly well-ordered trimers (9–15). In addition, we and others have derived ways to purify the native-like soluble HIV Env trimers from other isoforms using positive or negative selection methods to improve further the homogeneity of the Env candidate immunogens (16, 17). This conformer selection is not possible with membrane-bound trimers expressed by genetic means. We reason that expressing our stabilized NFL trimers anchored to the natural HIV TM might generate a more relevant mimic of the native spike as others have recently demonstrated using alternative HIV Env trimeric platforms (18–21). And, because the NFL Env is stabilized to a degree beyond the natural stability of native Env, the cell-surface expressed trimers might stay conformationally relevant for longer *in vivo*. Moreover, restoring the membrane occludes the unnaturally exposed protein base soluble trimers display following truncation before the MPER.

Previously, we demonstrated that furin cleavage of full-length native Env is required for membrane-bound spikes to display a favorable antigenic profile, consistent with the pre-fusion conformation of native Env and consistent with the similar requirement needed to generate well-ordered SOSIP trimers (22, 23). Overcoming the requirement of furin cleavage would be preferable for genetic expression of Env trimers, eliminating the need to co-express exogenous furin to ensure complete cleavage, a prerequisite for a well-ordered natural HIV Env or SOSIP conformation. For this reason, the NFL design and subsequent stabilized developments should be ideal for genetic expression where it is not possible to selectively purify well-ordered trimers by negative or positive selection (7, 9, 10, 14, 16, 24).

Here, we extend the stabilized NFL design to generate membrane-bound NFL trimers following genetic expression from both DNA and mRNA. For this, we follow two design strategies: either we tethered the NFL.664 trimer to the HIV TM via a second short flexible linker. Or, alternatively, we incorporate the full MPER region into our soluble trimer design, restoring the full Env ectodomain, and followed by the natural HIV TM and cytoplasmic tail (CT). In both soluble SOSIP and NFL trimers, the MPER was removed to avoid trimer aggregation and misfolding (25, 26). In our second design, we reasoned that it might be possible to restore this conserved, broadly neutralizing determinant in the membrane context without detriment to the native-like properties of these novel trimers expressed on the cell surface, which we have demonstrated *in vitro* and *in vivo* by elicitation of autologous tier-2 neutralizing serum antibody responses in immunized rabbits with three HIV strains. These are important developments for genetic expression of native-like Env trimers in animals and in humans as vaccine candidates.

## Materials and methods

### Design of the membrane-bound HIV Env NFL.711 trimer constructs

The Env sequences to generate NFL.711 constructs were derived from the following sequences stored at the GenBank sequence databank (1086c_FJ444395; JR-FL_U63632; BG505_DQ208458; 16055_EF117268). The cell surface NFL.711 Env protein design strategy included the replacement of the natural HIV leader sequence for the CD5 leader sequence in the plasmid DNA constructs or for the HLA-DR leader in the mRNA constructs. The four-residue furin cleavage site REKR (residues 508–511 of HIV Env gp140) were replaced by a flexible linker (G_4_S)_2_ following the NFL design. We, then, utilized two separate design strategies to link the gp140 residue D664 to the natural HIV transmembrane domain (TM). In the first strategy, we used a second small linker (G_4_S) to span the gap between residue D664 and residue K683 of the gp41 domain. In the second strategy, we restored the natural membrane proximal external region (MPER) from residue D664 to K683. Both design strategies are followed by the natural HIV TM (residues L684 to S703) and a truncated cytoplasmic tail (CT). The CT was truncated at residue G711 before the endocytosis signal to increase retention of membrane-bound trimeric Env proteins. The CT truncation at residue G711 is what gives the name to the membrane bound construct NFL.711. To all these general design modifications we then added individual stabilizing modification for each HIV Env as shown in **Supplementary Figure S1** in **Supplementary Material** following stabilization cassettes previously used for our soluble stabilized NFLs (9, 10).

### Generation and purification of soluble HIV Env NFL.664 trimers

Soluble trimers (for ELISAs, immunizations and EMPEM) were derived from the following GenBank databank sequences (1086c_FJ444395; JR-FL_U63632; BG505_DQ208458; 16055_EF117268) to generate stabilized NFL.664 (TD CC+) trimers as described in the past (9, 10). In brief, modified gp140 NFL genes were ordered from GenScript and cloned into a kanamycin resistant CMVR vector (NIH). A CD5 leader sequence was added at the N-terminus of the *env* NFL gene to allow secretion of the trimeric proteins into the supernatant. HEK 293F cells were transfected using fectin and after four days of incubation at 37°C in a rotating incubator, supernatants were collected and filtered. Supernatants were loaded overnight onto a lectin agarose column and affinity purified. Following lectin purification, trimers were further purified by size exclusion and negative selection if needed (16).

### Transfections of envelope glycoproteins from plasmid DNA and mRNA

A day before the transfection, approximately 8 x 10^6^ HEK 293T cells were seeded in a 150 mm tissue culture dish in DMEM, 10% FBS and 1% pen-strep buffer. The next day, plated cells were transfected with DNA plasmids encoding 1086c, BG505, JR-FL and 16055 NFL.711 constructs. DNA was mixed in Opti-mem buffer (Invitrogen) with the transfection reagent Fugene6 at 1:3 ratio with 5 µg of plasmid DNA per 1 x 10^6^ cells and incubated at RT for 20 minutes. The plasmid DNA/Fugene6 mix was then added dropwise to plated cells. Cells were incubated at 37°C and collected 48–72 hours after the transfection for FACS analysis.

For the mRNA transfections, plated HEK 293T cells were harvested and aliquoted in four separate wells in a 96 well-plate at a density of 3 x 10^6^ cells per well. Then, 5 µg of mRNA encapsulated in LNPs were dropped directly on to the cells without any transfecting reagent. The cells were incubated in the 96-well plate for 3 hours at 37°C to facilitate the transfection maximizing the contact between cells and the LNPs. Finally, cells were transferred to a 100 mm culture dish where they were cultured for 48 hrs. After 2 days the cells were harvested and prepared for flow cytometry analysis.

### mRNA design and production

The amino acid sequence of 1086c_FJ444395; JR-FL_U63632; BG505_DQ208458; 16055_EF117268 were obtained as above. The sequences underwent codon optimization and GC enrichment using our proprietary algorithm to improve expression and reduce potential immunogenicity of the *in vitro* transcribed mRNA. The codon optimized sequences were gene synthetized by GenScript with an optimized secretion signal, cloned into our proprietary *in vitro* transcription template containing an optimized T7 promoter, 3’UTR, 5’UTR and a 100-adenine tail. The nucleoside modified mRNA sequences were prepared using the MegaScript transcription kit (ThermoFisher Scientific), co-transcriptionally capped using the CleanCap™ system (TriLink Biotechnologies) and purified using a modified cellulose base chromatography method, precipitated, eluted in nuclease free water, and quantified using the NanoDrop One system. Length and integrity were determined using denaturing agarose gel. Endotoxin content was measured using the GenScript Toxisensor chromogenic assay, and values were below detection levels (0.1 EU/mL). mRNA was frozen at -20°C until formulation.

### Production and characterization of mRNA-LNP vaccines

The LNP formulation used in this study is proprietary to Acuitas Therapeutics; the proprietary lipid and LNP composition are described in US patent US10,221,127. The RNA-loaded and empty particles were characterized and subsequently stored at −80°C at an RNA concentration of 1 μg/μl and total lipid concentration of 30 μg/μl. The hydrodynamic size, polydispersity index (PDI) and zeta potential of mRNA-LNPs were measured using a Zetasizer Nano ZS90 (Malvern Instruments, Malvern, UK). The mRNA encapsulation efficiency of LNP were determined using a modified Quant-iT RiboGreen RNA assay (Invitrogen). The mean hydrodynamic diameter of mRNA-LNPs was approximately 80 nm with a polydispersity index of 0.02–0.06 and an encapsulation efficiency of approximately 95%. Endotoxin levels were determined using the Limulus Amebocyte Lysate (LAL) chromogenic assay found to be <0.5 endotoxin unit (EU)/mL.

### Animal immunization and sampling

New Zealand white female rabbits were purchased and housed at ProSci, Inc. Six groups of five rabbits were immunized bilaterally over the hips with a total volume of 200 µL per site. Briefly, the mRNA LNPs were kept frozen at -80°C for long term storage. The day of inoculation the LNPs were allowed to thaw and diluted in PBS for inoculation. Animals received 5 µg of mRNA per dose. Animals that received soluble protein received 30 µg of protein adjuvanted with Adjuplex (20% v/v) (Sigma-Aldrich).

### Antigenicity of the cell surface NFL.711 proteins and serum IgG binding by FACS analysis

HEK 293T cells expressing NFL.711 trimeric proteins were harvested 72 hours after DNA plasmid or mRNA LNP transfection by pipetting. Cells were filtered through a 0.7 μm filter. Cells were washed twice with FACS staining buffer and aliquoted to a 96 round-well plate (approximately 1 to 3 million cells per well). In a separate 96 well plate, selected antibodies were added at starting concentration of 25 µg/mL and serially diluted five-fold down the plate six times (Vol. 100 µL per well). The antibody dilutions were transferred from the antibody dilution plate to the plate containing the harvested cells and mixed. The antibody/cells mix was incubated at 4°C for one hour on a rotating platform or lightly shaking so that the cells would not settle. Then, the cells were washed three times with FACS buffer. Secondary anti-human PE conjugated antibody was added at a 1:200 dilution (vol. 100 µL per well). Cells were incubated with a secondary antibody for 1 hour at 4°C. Cells were washed three times with FACS buffer to remove excess or unbound antibodies. The samples were then run in a Novacyte Flow cytometer. Mean fluorescence Intensity (MFI) values were annotated corresponding to each antibody dilution binding to cells expressing the membrane-bound NFL trimeric proteins and plotted as a datapoint on that antibody dilution series to make the binding curves. These experiments were done several times and two of the experiments are reported in the main figures with standard error of the means. For the serum IgG samples used for the binding versus correlation experiments. We used an anti-rabbit PE conjugated antibody (Biolegend). Total IgG starting concentrations were 100 µg/mL and serially diluted six times to get a binding curve.

### Pseudovirus production

HEK293 T cells were seeded 24 hours before the transfection at a density of 3 x 10^6^ per T-75 flask. Plasmids encoding Env corresponding to the HIV/SIV strains to be tested were co-transfected in HEK 293T cells with an Env-deficient backbone plasmid (pSG3ΔENV) using Fugene6 at a 1:3 DNA: Fugene6 ratio, 15 µg of the backbone and 5 µ of Env per T-75 flask. Cell media containing pseudoviruses were harvested 72 hours after the transfection and stored at -80°C. Pseudoviruses are titrated prior to use in the neutralization assay to determine the appropriate dilution of the virus and the need for dextran. Dextran increases infectivity and is used with viruses that produce lower luciferase signals.

### Neutralization assay

Neutralization activity of the serum and purified IgG samples were carried out in TZM-bl target cells as previously described (27). Briefly, 1x10^4^ TZM-bl cells were plated per well in a tissue culture 96 well plate the day before (vol. 100 µL of complete DMEM). In a separate U-bottom plate, the samples (serum or IgG) were prepared and diluted accordingly, then distributed to assay plates where they were mixed with the viruses tested. The sera were initially diluted 1:10 and, for purified IgG samples the starting concentration of purified IgG was 2000 µg/mL which approximately compares to a 1:5 dilution of the serum in potency. These quantities (1:10 and 2000 µg/mL) are the final concentrations accounting for the 5-fold dilution with the virus when 40 µL of virus is added to the 10 µL of serum or IgG sample per well. These assay plates were then incubated for 1hr at 37°C to allow the antibodies in the serum or IgG samples to interact with the pseudoviral particles. The media of the tissue culture plates containing the TZM-bL cells was aspirated carefully as to not disturbed the adhered cells (alternatively, you could add the 2x10^4^ cells per well the day of the assay). Following the aspiration of the media, the serum antibody-virus mix was transferred to the TZM-bL cell culture plates and incubated for 1hr at 37°C to facilitate viral infection. After that incubation, fresh media was added to the cells (on top of the antibody virus mix) and the cells were incubated for 48 hours. Media was removed from the cell plates after 48 hours. The cells were lyzed for 20 min in a rocking platform. Then cell plates containing the lysates were taking to a NEO2M (BioteK) instrument where the luciferase substrate was added. Luciferase signals are provided in RLUs then converted to serum neutralization activity and reported as ID50s, inhibitory serum dilution that resulted in 50% of inhibition of viral entry. Similarly, the purified serum IgG neutralization assays were reported as IC50s, inhibitory IgG concentration that mediates 50% of inhibition of viral entry.

### Purification of IgG from serum

In a 12 mL conical tube, 1 mL of Protein A slurry was added and washed with PBS twice by centrifugation at 3000 x *g* and carefully aspirating the supernatant to remove any traces of ethanol or other preservatives. After washing the agarose beads, 5 ml of PBS and 1 mL of serum sample were added and mixed; then, rocked at RT for 1 hour so that the IgG would bind the Protein A. On a rack, an equal number of disposable columns as serum samples were prepared for purification. The diluted sample-agarose mix was added to a column by pipetting the entire contents of the tube into the column. The sample was allowed to flow through the column while retaining the agarose beads containing the bound IgG. The column was washed by adding 5 mL of 0.5 M NaCl PBS to the column. The bound IgG was eluted by adding 5 mL of IgG elution buffer (Thermos Scientific Ref. 21009) and the acidic pH neutralized by adding 1 M Tris, pH9 (100 µL/ml of eluate). The eluate containing the IgG was buffered exchanged to PBS in an Amicon Ultra centrifugal filter device (30K) and adjusted to a concentration of 10 mg/mL to normalize the IgG sample concentrations in preparation for the neutralization assay.

### Immunoprecipitation and gel electrophoresis of membrane bound NFL trimers

Approximately 8 x 10^6^ HEK 293T cells were seeded in a 150 mm tissue culture dish in DMEM, 10% FBS and 1% pen-strep buffer. The next day, plated cells were transfected with DNA plasmids encoding 1086c, BG505, JR-FL and 16055 NFL.711 constructs. Cells were collected 72 hours after the transfection and pelleted at 500 x *g* for 5 min. Then, the cells expressing the cell surface trimeric proteins were washed once with PBS and allocated to 4 different tubes in 1 mL of 0.5% Triton X-100 PBS. Cells were incubated in 0.5% Triton X-100 PBS to dislodge the cell membrane and release the membrane proteins. We then pelleted the cell debris by centrifugation at 12000 x *g* and carefully collected the supernatant containing the solubilized trimeric proteins. We added VRC01, PGT145, 10E8 and F105 to a final concentration of 25 µg/mL separately to each cell aliquot and let it incubate for 1 hour at 4°C. We added 40 µL of Protein A beads to the sample and rock at 4°C for 30 mins. Then we washed the protein A beads three times with 0.5 M NaCl PBS to remove unbound proteins. Beads were pelleted by centrifugation and excess supernatant removed. At this point, the gel loading buffer was added, subjected the samples at 100°C for 3 mins to denature the proteins and then the denatured samples were loaded into a Bolt™ 4–12% Bis-Tris Plus gel (Invitrogen ref. NW04120BOX) and run at 150 V for 35 min.

### ELISA binding assays

The day before the assay, we coated 96 well plates with 2 µg/mL (Vol 100 µl per well) of the specific soluble antigen, soluble NFL trimeric proteins matching the immunogen received by the animal whose sera samples are being tested. The next day, after washing the coated plates with PBS, we added blocking buffer (Vol. 300 µL per well, 2% milk, 5% FBS PBS) and incubated the plates at RT for 2 hours. The plates were then washed three times with PBS 0.05% Tween by adding 300 µL of wash buffer to each well. Then, the serum samples were added and serially diluted (starting from 1:50 six times 5-fold dilutions). After an hour incubation at 37°C, we washed the plates three times with wash buffer. Secondary antibody (goat anti-rabbit Fc fragment) was added (100 µL per well) at a 1:2000 dilution. The plates were incubated at 37°C for 30 min and then washed 3 times. Then the substrate (3,3’,5,5’ -tetramethylbenzidine (TMB) was added (100 µL per well). Each plate was allowed to develop for 2 min and then the reaction was stopped by adding (100 µL per well) of 0.16 M H_2_SO_4_ to each well. Colorimetric reactions were read at an OD wavelength of 450nm in a BioteK NEO3M spectrophotometer.

### Intracellular staining of transfected HEK 293T cells

HEK 293T cells that were transfected with 5 µg of NFL.711 encoding mRNA LNPs following the method described in this section. After 48 hours in culture at 37°C, the protein transport inhibitor containing Brefeldin A (BD, GolgiPlug) was added at a concentration of 1 µL/mL of cell culture. The cells were incubated in the presence of the inhibitor for 3 hours at 37°C. Cells were harvested by gently scraping them off and placed in FACS staining buffer containing Fc block receptor (FcγII/III receptors, BD Fc Block Cat. No. 553142) for 15 min at 4°C. Cells were washed and permeabilized with the fixation/permeabilization solution (BD, Cytofix/Cytoperm kit, Cat. No. 554714). Permeabilized cells were washed twice with the kit provided wash buffer before proceeding with antibody staining.

### EMPEM analysis

IgG was isolated as described above (Purification of IgG from Serum). Papain (Sigma Aldrich) was used to digest IgG to polyclonal antigen-binding fragments (polyFab). Trimer-polyFab complexes were prepared and incubated overnight by mixing 15 µg of 1086c or JR-FL NFL.664 trimer with 1 mg of Fab mixture (containing Fc and residual papain). On the next day, the complexes were purified using a Superdex 200 Increase 10/300 GL gel filtration column (Cytiva). Purified complexes were concentrated and diluted to a final concentration of 0.03 mg/mL, which were adsorbed on glow-discharged carbon coated copper mesh grids and stained with 2% (w/v) uranyl formate. Electron microscopy images were collected on an FEI Tecnai TF20 equipped with an TVIPS TemCam F416 CMOS camera (120 keV, 1.68 Å/pixel) using the Leginon automated collection software, and processed using Relion 3.0 following standard 2D and 3D classification procedures. UCSF Chimera was used to generate the composite maps, and the representative maps with identified epitopes have been deposited to the Electron Microscopy Data Bank.

### Glycan profiling of NFLs

DeGlyPHER (28) is used to ascertain site-specific glycan occupancy and processivity on the examined glycoproteins.

### Proteinase K treatment and deglycosylation of Env samples

HIV Env glycoprotein (when membrane bound, denatured in 6 M urea) was exchanged to water using Microcon Ultracel PL-10 centrifugal filter. Glycoprotein was reduced with 5 mM tris(2-carboxyethyl)phosphine hydrochloride (TCEP-HCl) and alkylated with 10 mM 2-Chloroacetamide in 100 mM ammonium acetate for 20 min at room temperature (RT, 24°C). Initial protein-level deglycosylation was performed using 250 U of Endo H for 5 µg trimer, for 1 h at 37°C. Glycorotein was digested with 1:25 Proteinase K (PK) for 30 min at 37°C. PK was denatured by incubating at 90°C for 15 min, then cooled to RT. Peptides were deglycosylated again with 250 U Endo H for 1 h at 37°C, then frozen at –80°C and lyophilized. 100 U PNGase F was lyophilized, resuspended in 20 µl 100 mM ammonium bicarbonate prepared in H_2_^18^O, and added to the lyophilized peptides. Reactions were then incubated for 1 h at 37°C, subsequently analyzed by LC-MS/MS.

### LC-MS/MS

Samples were analyzed on a Fusion Lumos mass spectrometer. Samples were injected directly onto a 25 cm, 100 μm ID column packed with BEH 1.7 μm C18 resin. Samples were separated at a flow rate of 300 nL/min on an EASY-nLC 1200 UHPLC. Buffers A and B were 0.1% formic acid in 5% and 80% acetonitrile, respectively. The following gradient was used: 0–25% B over 120 min, an increase to 40% B over 40 min, an increase to 100% B over another 10 min and 10 min at 100% B for a total run time of 180 min. Column was re-equilibrated with solution A prior to the injection of sample. Peptides were eluted from the tip of the column and nanosprayed directly into the mass spectrometer by application of 2.8 kV at the back of the column. The mass spectrometer was operated in a data dependent mode. Full MS1 scans were collected in the Orbitrap at 60,000 resolution. The most abundant ions per scan over a set cycle time of 3 s, were selected for HCD at 30 NCE and MS/MS scans were collected in the Orbitrap at 7,500 resolution. Dynamic exclusion was enabled with exclusion duration of 10 s and ions with charge state +2 to +7 were included.

### Data processing

Protein and peptide identification were done with Integrated Proteomics Pipeline (IP2). Tandem mass spectra were extracted from raw files using RawConverter (29) and searched with ProLuCID (30) against a database comprising UniProt reviewed (Swiss-Prot) proteome for Homo sapiens (UP000005640), UniProt amino acid sequences for Endo H (P04067), PNGase F (Q9XBM8), and Proteinase K (P06873), amino acid sequences for the examined proteins, and a list of general protein contaminants. The search space included no cleavage-specificity. Carbamidomethylation (+57.02146 C) was considered a static modification. Deamidation in presence of H_2_^18^O (+2.988261 N), GlcNAc (+203.079373 N), oxidation (+15.994915 M) and N-terminal pyroglutamate formation (–17.026549 Q) were considered differential modifications. Data was searched with 50 ppm precursor ion tolerance and 50 ppm fragment ion tolerance. Identified proteins were filtered using DTASelect2 (31) and utilizing a target-decoy database search strategy to limit the false discovery rate to 1%, at the spectrum level (32). A minimum of 1 peptide per protein and no tryptic end per peptide were required and precursor delta mass cut-off was fixed at 15 ppm. Statistical models for peptide mass modification (modstat) were applied. Census2 (33) label-free analysis was performed based on the precursor peak area, with a 15 ppm precursor mass tolerance and 0.1 min retention time tolerance. “Match between runs” was used to find missing peptides between runs. Data analysis using GlycoMSQuant (28) was implemented to automate the analysis. GlycoMSQuant summed precursor peak areas across replicates, discarded peptides without NGS, discarded misidentified peptides when N-glycan remnant-mass modifications were localized to non-NGS asparagines and corrected/fixed N-glycan mislocalization where appropriate.

### Proteomics data repository

The proteomics data have been submitted to the UCSD MassIVE repository (MSV000094915). The proteomeXchange ID is PXD052769.

## Results

### Design of membrane-bound HIV Env NFL trimers for genetic expression

**Figure 1:**
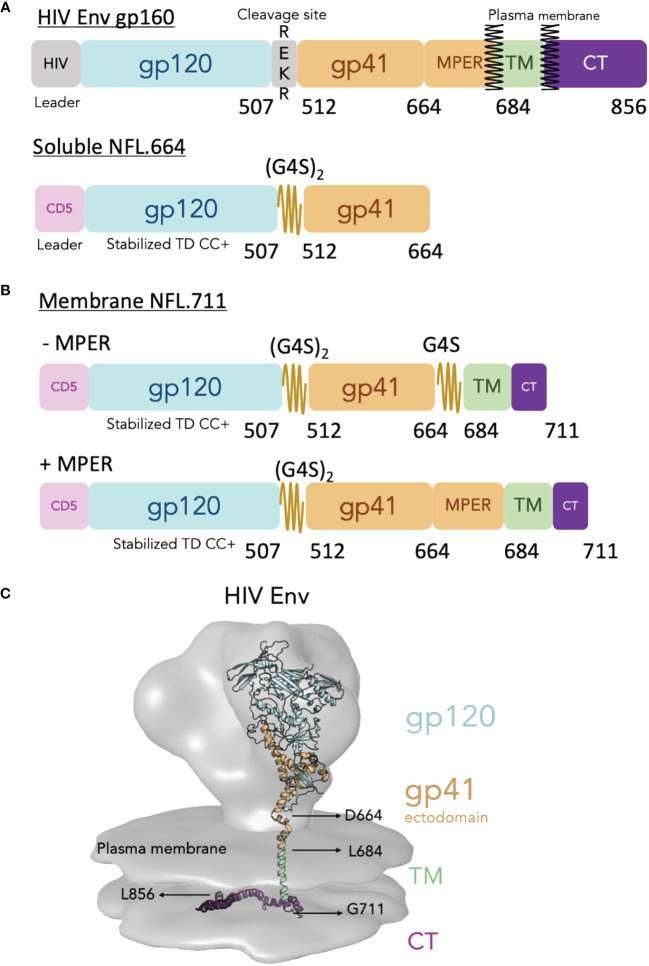
Design of the cell surface single chain HIV Env mimic NFL.711. (**A**, top) Schematic of the HIV Env gp160 precursor displaying the surface subunit gp120 (blue) and the membrane subunit gp41 ectodomain (brown), the transmembrane domain (TM, green) and the cytoplasmic tail (purple). The natural HIV Env cleavage site (_508_REKR_511_) between gp120 and gp41 is shown. (A, bottom) Schematic of the soluble Env NFL.664 construct. The heterologous CD5 leader (pink) and the engineered linker (2xG4S) are indicated. Not shown in the diagram for simplicity but included in the stabilized NFL design are a cassette of stabilizing mutations designated as “TD CC+” (See **Supplementary Figure S1** in **Supplementary Materials**). **(B)** Schematic of the membrane-bound NFL.711 constructs without (-MPER, top) and with (+MPER, bottom) the membrane proximal external region (MPER). The NFL.711 construct follows a similar stabilization design as the soluble NFL.664 trimer (TD CC+, see **Supplementary Figure S1** in **Supplementary Materials**). The (-MPER) design includes a second short G4S linker in place of the MPER. **(C)** Reconstruction of the membrane-bound HIV Env showing the cryo-EM tomography density of the natural HIV Env (gray) with an underlying-colored ribbon crystal structure that indicates the different regions of Env. The following structures and EM densities were used to make this reconstruction (16055 NFL TD CC+ PDB ID 5UM8, MPER and TM regions PDB ID 6E8W, CT region PDB ID 7LOH and for the EM Spike density EMD-5272).

To design our cell-surface membrane-bound NFL trimers, that from hereon we will refer to as NFL.711 (number refers to the last residue of HIV Env incorporated in the construct), we introduced several modifications from the natural Env sequence (see **Figure 1A** and **Supplementary Figure S1** in **Supplementary Material**). To begin, we used a heterologous CD5 leader sequence in place of the natural HIV signal sequence to increase Env trimer expression (34). Next, we codon optimized the natural HIV Env sequences to eliminate the RRE regulatory stem loop and render Env mRNA export to the ribosomes independent of *rev* co-expression. In addition, we modified natural Env coding for residues that incorporate our cassette of stabilizing mutations developed to generate homogeneous soluble HIV Env NFL trimers (**Figure 1A** and **Supplementary Figure S1** in **Supplementary Material** for Env mutation details). We reasoned that this successful stabilizing strategy for the soluble NFL.664 trimers would transfer well to membrane-bound, cell-surface NFL trimer expression (9, 10, 35–37). As a test case, we selected the African HIV-1 1086c viral Env sequence to design membrane-bound NFL trimers because previously we had successfully used this Env strain to improve the proportion of well-ordered soluble trimeric Env proteins expressed following transient expression (10). We retained features of the original NFL design that include a flexible linker (G_4_S)_2_ between the gp120 and gp41 subunits replacing the natural furin cleavage site, REKR, at the C-terminus of gp120, thus, rendering the trimer cleavage-independent (**Figure 1A**) (7). We used two different strategies to link the Env ectodomain to the TM. In the first design, we employed a second small G_4_S linker to ‘fill the gap’ between the C-terminus of our NFL Env at residue 664 and the natural HIV TM beginning at residue 684. This linker bridging Env residues 664 to residue 684 (original HXBc2 numbering) facilitates the linkage to the TM in the absence of the natural MPER (**Figure 1B**, top). Our second strategy restored the full natural MPER region (665–683) followed by the natural TM (**Figure 1B**, bottom). Finally, to increase cell-surface expression, we truncated the natural long Env CT at residue 711, removing the normal endocytosis signals that can limit Env on the plasma membrane (38, 39) (**Figure 1B**). These modifications should result in trimers embedded in the plasma membrane from cells transiently transfected with genetic material both *in vitro* and *in vivo* (**Figure 1C**).

### Expression and antigenicity of membrane-bound NFL Envs on the cell surface following DNA transient transfection is consistent with a near-native conformation

**Figure 2:**
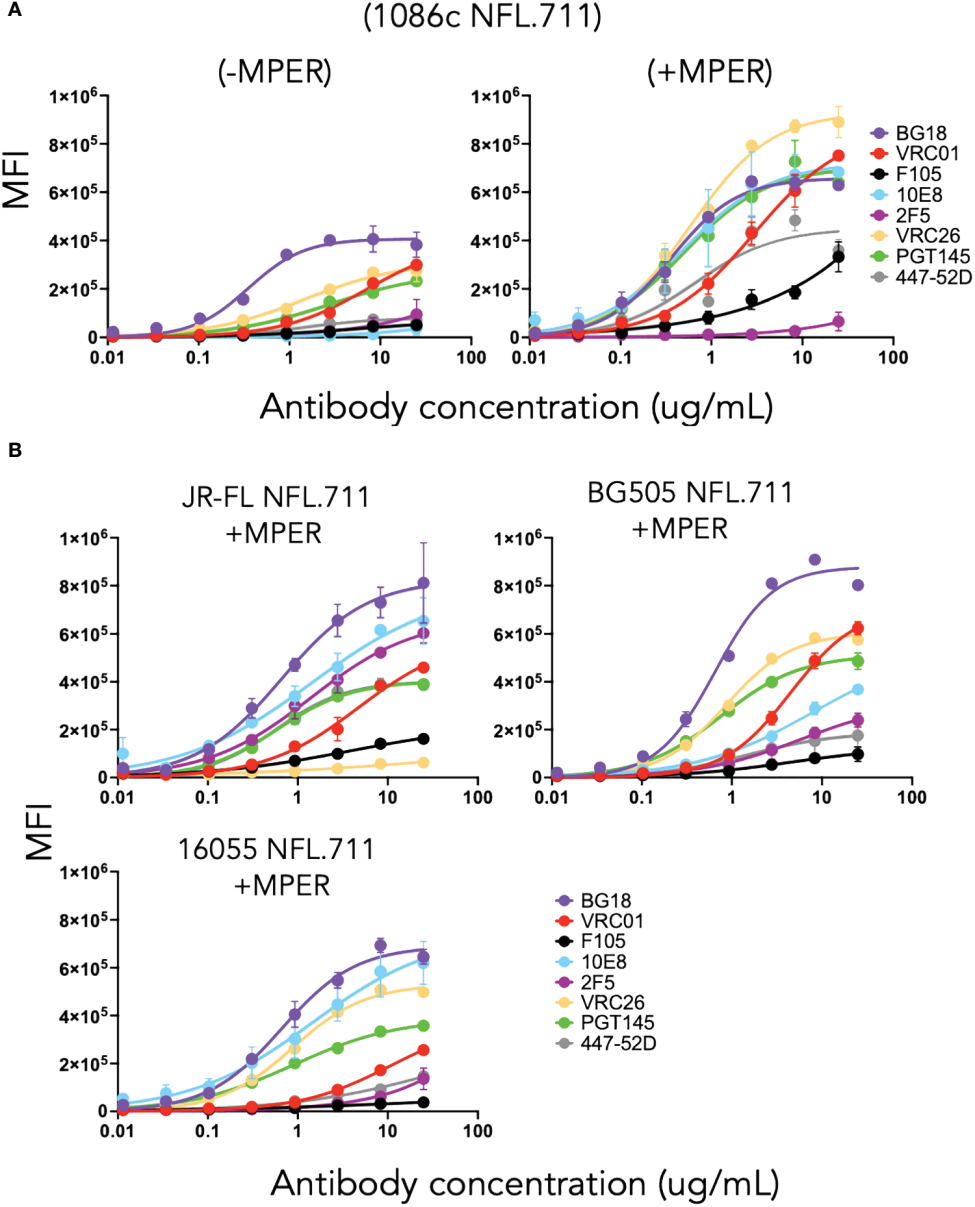
Antigenicity by flow cytometry on cells transfected with plasmid DNA encoding NFL.711 constructs. **(A)** Test case membrane-bound 1086c NFL.711 trimers expressed from plasmid DNA. Selected antibodies were used (25 µg/mL starting concentration, serially dilute 5-fold) to determine binding to trimeric NFLs expressed on the cell surface of HEK293 T cells by flow cytometry. Here, we compare the (-MPER) construct, lacking the membrane proximal external region, (left) with the (+MPER) (right) design on the 1086c Env. The datapoints represent the average MFI levels and SEM resulting from two independent experiments expressing NFL.711 proteins. Transfected cells were stained with the broadly neutralizing antibodies bnAbs (BG18, PGT145, VRC01, 10E8 and 2F5, the latter not neutralizing for the 1086c HIV strain) and the non-broadly neutralizing antibodies (F105 and 447–52D). The relative binding of these antibodies alludes to the conformational state of the trimeric proteins on the cell surface. **(B)** Similarly, as above, we tested the antigenicity of three additional NFL.711 constructs (+MPER) derived from a clade B, JR-FL, a clade A, BG505 and a clade C, 16055 Env sequences that extend the applicability of our cell surface design.

Initially, we sought to determine if the newly designed 1086c NFL.711 construct generated membrane-bound trimeric Env on the cell surface of transiently transfected HEK 293T cells. By flow cytometry, we assessed cell-surface Env expression and conformational state of the expressed proteins by ‘antigenic profiling’. Selected HIV antibodies were used for this analysis: PGT145 and VRC26, broadly neutralizing antibodies (bnAb) that exclusively recognize a quaternary epitope of native Env (40–42); VRC01, a CD4 binding-site bnAb (43); BG18, a bnAb that targets the N332 glycan supersite; and two MPER-directed bnAbs, 10E8 and 2F5 (44, 45), to validate that their respective target sites are present and accessible in our trimeric membrane-bound proteins. In addition, we selected two non-neutralizing antibodies (unable to neutralize ‘tier-2’ clinical isolates), F105 (CD4bs-directed) (46) and 447–52D (V3-directed) (47), that recognize their epitopes on undesirable open forms of Env and cannot access their epitopes on well-ordered and well-folded trimeric spikes. A desirable antigenic outcome would retain binding of the bnAbs while minimizing the binding of F105 and 447–52D antibodies, indicating that the expressed trimeric proteins on the cell membrane adopt a well-ordered native-like conformation. Following transient expression from plasmid DNA encoding 1086c NFL.711 constructs, we analyzed both 1086c variants, lacking or incorporating the MPER, named “-MPER” (lacking the MPER) and “+MPER” (possessing the natural MPER). We determined that both trimer types were expressed on the cell surface of transfected HEK293 T cells with the (+MPER) variant having higher levels of expression as assessed both by flow cytometry and by gel electrophoresis after antibody immunoprecipitation (**Figure 2A** and **Supplementary Figure S2** in **Supplementary Material**). Both constructs were efficiently recognized by the bNAbs PGT145, VRC26, BG18 and VRC01 and minimally by the non-neutralizing F105 and 447–52D mAbs, Their antigenic profiles indicated that the majority of the expressed trimers expressed on the cell surface retained a native-like conformation (**Figure 2A**). Consistent with design criteria, the MPER-directed bNAb, 10E8, recognized only the +MPER construct but not the -MPER trimer construct because the domain was genetically removed in the latter design (**Figure 2A**, right and **Supplementary Figure S2** in **Supplementary Material**). In agreement with its inability to neutralize clade C strains, the MPER-directed but clade B-restricted 2F5 bNAb did not bind the +MPER clade C 1086c NFL.711 trimers. Based on these encouraging results, we expanded our cell-surface design NFL.711 (+MPER) to three other Env strain sequences, namely, BG505, JR-FL and 16055 (Clades A, B and C, respectively) to determine more general applicability. The new three constructs followed the same general design as done for 1086c (+MPER) since this design generated higher expression of surface Env and contained additional bNAb target sites. Following production of plasmid DNA and transient transfection, we then assessed expression by flow cytometry and determined the level of binding by the same set of antibodies. This next set of membrane-bound trimers were well recognized by the bNAbs PGT145, BG18, VRC01 and by the MPER-directed 10E8 but minimally recognized by the non-neutralizing antibodies F105 and 447–52D (**Figure 2B**). The 2F5 antibody selectively recognized membrane-bound NFL trimers corresponding to Envs from HIV strains for which it neutralizes: BG505 and JR-FL. The antigenic profiling suggested that the membrane-bound trimeric proteins retained a predominantly native-like trimer conformation. Additionally, we tested the binding of the V3-N332-glycan targeting antibody 2G12, the MPER-targeting 4E10 and the fusion peptide-directed VRC34 antibodies against the JR-FL and 1086c proteins. Both 4E10 and 2G12 showed significant binding while VRC34 did not (**Supplementary Figure S3**).

In parallel, we expressed the membrane-bound proteins in HEK 293T cells and extracted the cell-surface Env NFL.711 proteins with a 0.5% triton solution to solubilize the membrane-bound trimers. We used a panel of four antibodies, PGT145, 10E8, VRC01 and F105 to immuno-precipitate the Env proteins and performed polyacrylamide SDS-gel electrophoresis to assess both conformation and relative level of expression (**Supplementary Figure S2** in **Supplementary Material**). This analysis was consistent with that of the flow cytometry, indicating that all four Env strains were largely expressed in a native-like conformation recognized by the quaternary antibody PGT145 (**Supplementary Figure S2** in **Supplementary Material**).

### Cell-surface NFLs are well-expressed from mRNA lipid nanoparticles *in vitro* and retain a native-like conformation

**Figure 3:**
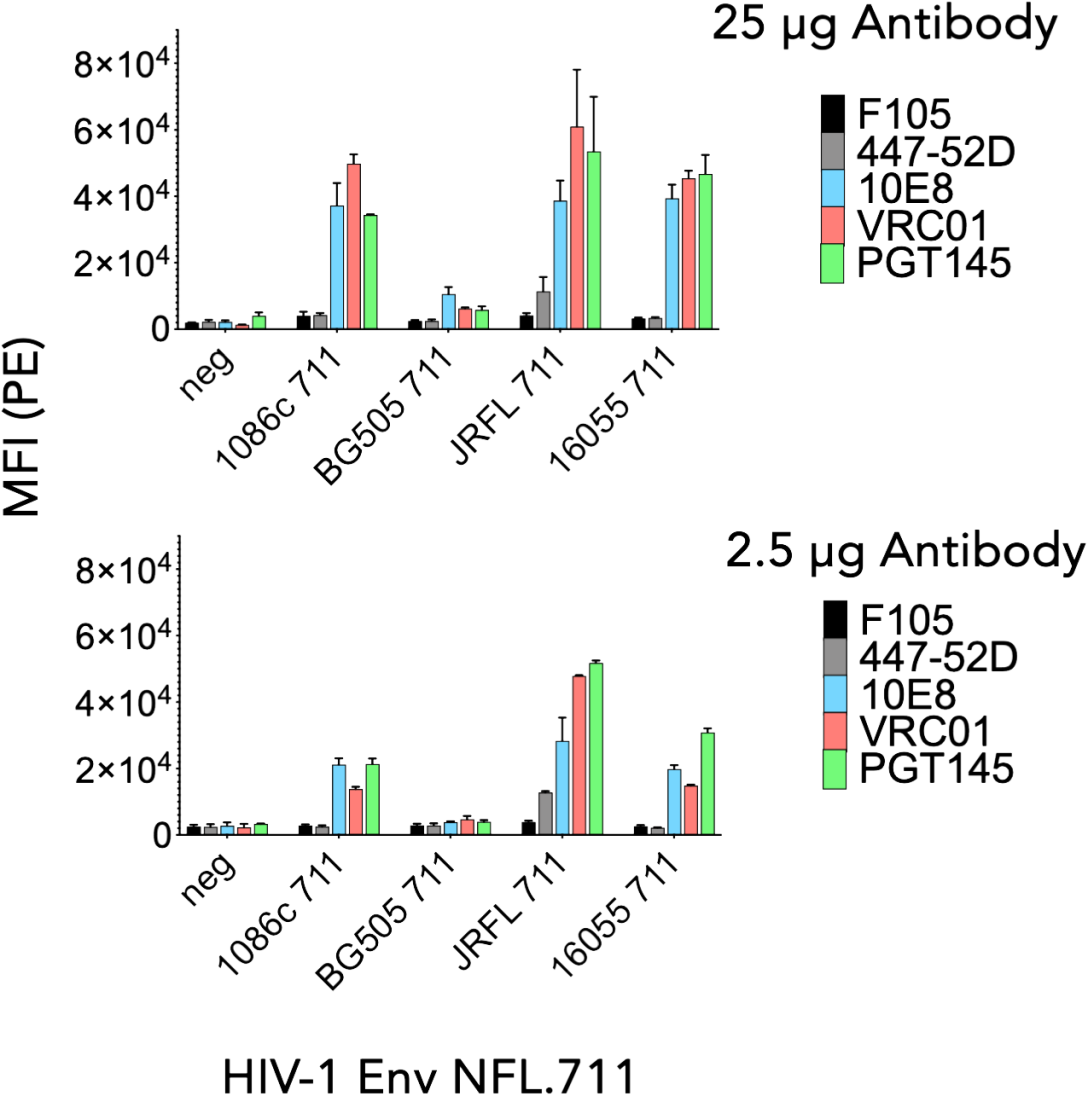
Antigenicity by flow cytometry of NFL.711 proteins expressed from mRNA LNPs. Bars represent MIF values with SEM corresponding to the binding of an antibody to a trimeric membrane-bound Env protein expressed on the cell surface of the HEK293 T cells transfected with mRNA LNPs encoding the NFL.711 (+MPER) constructs. Here, we show the MFI values as bars with SEM corresponding to two separate experiments at two concentrations of the antibodies, 25 μg/ml (Top) and 2.5 µg/ml (bottom).

To determine *in vitro* expression of the four Env NFL.711 trimers from mRNA we generated 1086c, BG505, JR-FL and 16055 NFL.711 (+MPER) encoding, nucleoside-modified mRNA encapsulated lipid nanoparticles. These constructs are identical at the amino acid level to those encoded by the DNA plasmids described in the previous section, however, the coding nucleotide sequences were codon-optimized appropriately to enhance expression from mRNA (see Methods). Following transfection of HEK 293T cells with the mRNA lipid nanoparticles, we assessed Env trimer expression by FACS and antigenicity of the membrane-bound Env proteins. Following a similar approach to the antigenic characterization of the constructs expressed from plasmid DNA, we assessed binding of antibodies PGT145, VRC01, 10E8, F105 and 447–52D (40, 42, 43, 46, 48, 49) to determine the antigenic properties of the Env proteins expressed from mRNA. We used two antibody concentrations, 25 µg and 2.5 µg per ml for FACS analysis in two independent experiments. We plotted the data as bars representing the mean fluorescence intensity (MFI) with SEM corresponding to each antibody binding to a membrane-bound NFL trimer (**Figure 3**). The 1086c, JR-FL and 16055 NFL.711 proteins expressed robustly from mRNA LNPs based on PGT145, VRC01 and 10E8 bNAb recognition at both antibody concentrations 25 µg/mL and 2.5 µg/mL (**Figure 3**). Importantly, recognition by the non-neutralizing F105 and 447–52D antibodies was not distinguishable from controls (‘cells only’ negative controls), indicating that the trimeric proteins expressed on the cell surface from mRNA were well-ordered native-like Env trimers, in alignment with our observations from DNA plasmid transfections. In contrast, expression of BG505 NFL.711 trimers from mRNA LNPs was barely detectable at the highest antibody concentration tested (**Figure 3**, top). While the expression and binding results for the 1086c, 16055 and JR-FL constructs were comparable to those acquired from DNA plasmid transfections, the BG505 expression markedly differed (**Figures 2B**, **3**). We next investigated if a higher dose of the mRNA would increase BG505 NFL.711 expression. Accordingly, we performed a dose titration experiment where we transfected HEK 293T cells with increasing amount of mRNA 5, 10, 30 and 40 µg of mRNA LNPs encoding BG505 NFL.711. We then assessed BG505 NFL.711 trimer expression by FACS using 10E8 and F105 as the detecting antibodies, as 10E8 binds to a linear epitope on trimers and F105 will bind to open forms of Env, assuring us of detecting all forms of Env. We did detect higher BG505 NFL.711 Env expression with increasing amounts of mRNA as determined by increasing levels of 10E8 binding, however still relatively low expression even at 8 times the amount of mRNA transfected (**Supplementary Figure S4A** in **Supplementary Material**). BG505 NFL.711 expression was considerably lower than that of 1086c, JR-FL and 16055 NFL.711 based on the level of mean fluorescence intensity detected (**Figure 3**). Following cell permeabilization using the Cytofix/Cytoperm Fixation/Permeabilization kit, (BD Cat. No. 554714), we determined by intracellular flow cytometry antibody staining that much of BG505 Env was disproportionally retained in the cytoplasm while only a fraction was transported to the cell surface (**Supplementary Figure S4B** in **Supplementary Material**). For other Envs tested in this manner, the relative amount of 16055 and JR-FL Env trimers detected on the cell surface was larger than that of BG505 (**Supplementary Figure S4C** in **Supplementary Material**).

In sum, 1086c, JR-FL and 16055 NFL.711 membrane-bound trimers were well-expressed and generated predominantly well-ordered trimeric proteins on HEK293 T cells transfected with mRNA LNPs while BG505 Env was poorly expressed on the cell-surface.

### Membrane-bound NFL trimers are immunogenic following multiple inoculations with mRNA LNPs *in vivo*

**Figure 4:**
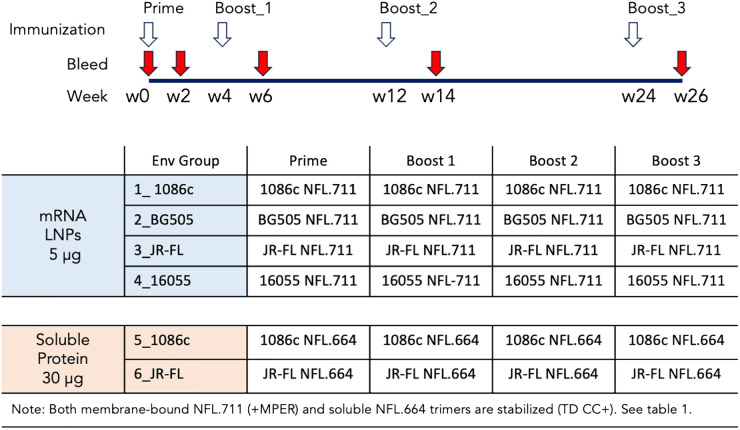
Immunogenicity schedule and immunogen regimens. Six groups of five rabbits were immunized 4 times following the immunogen regimens and the schedule shown. Colored in light blue are the groups of animals immunized with mRNA LNPs and colored in light orange the two groups that received soluble protein in adjuvant. The animals were immunized four times at 0, 4, 12 and 24 weeks as denoted with white arrows. Bleeds were collected at the time points specified in the figure with red arrows.

**Figure 5:**
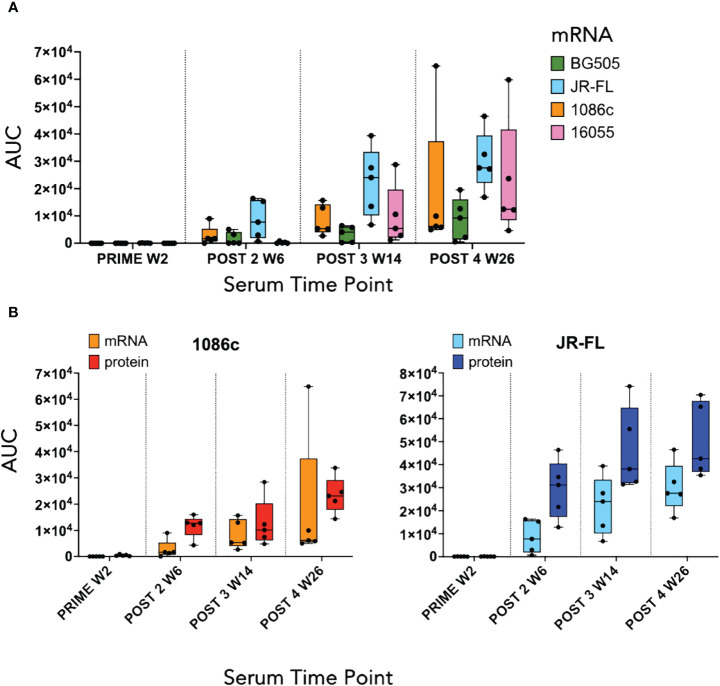
ELISA serum binding titers at the two-week timepoint following each immunization. **(A)** Box and whisker plot corresponding to serum binding titers of animals that received mRNA LNPs inoculations displaying individual area under the curve (AUC) values, minimum and maximum values, and the group median. Each color represents a different Env immunogen, orange for 1086c NFL.711, green for BG505 NFL.711, blue for JR-FL NFL.711 and pink for 16055 NFL.711 **(B)** Comparison of ELISA serum binding titers of animals that received either mRNA LNP inoculations or a HIV Env matched soluble protein control. Left graph shows data corresponding to animals immunized with 1086c NFL.711 encoding mRNA LNPs in orange or soluble protein in red. Likewise, on the right, ELISA titers are shown corresponding to animals that received JR-FL NFL.711 encoding mRNA LNPs in light blue or matched soluble protein in dark blue.

We next conducted a rabbit immunogenicity study testing mRNA LNPs encoding the trimeric membrane-bound NFL.711 proteins expressed *in vivo*. Four groups of five rabbits each were inoculated with 1086c, BG505, JR-FL and 16055 NFL.711 encoding mRNA LNPs four times at 0, 4, 12 and 24 weeks with 5 µg of mRNA via the intramuscular route (**Figure 4**). We selected 5 µg of mRNA as an adequate dose for rabbits following a SARS-COVID-2 mRNA-1273 vaccine study conducted in mice at NIH-VRC where they tested a low dose 0.1 µg vs a higher dose of 1 µg of mRNA where they concluded that 1 µg elicited a robust anti-SARS COVID-2 neutralizing antibody response in mice after just two incoculations. To compensate for the larger size of the rabbit we estimated that a 5-fold dose should suffice. To compare mRNA to soluble protein delivery we added two additional groups that received 25 µg of 1086c and JR-FL stabilized (TD CC+, see **Supplementary Figure S1** in **Supplementary Material**) NFL.664 soluble proteins in adjuvant, our standard protein dose for small animals (**Figure 4**). ELISA binding revealed that all immunogens generated antigen-specific serum antibody binding responses after four inoculations (**Figure 5**). Antibody binding responses varied in magnitude between the different mRNA immunogens and amongst animals. The group that received the mRNA LNPs encoding BG505 showed the lowest antibody responses. In contrast, groups that received mRNA LNPs encoding 1086c, JR-FL and 16055 showed higher serum binding antibody responses at all time points compared to BG505 (**Figure 5A**). When comparing soluble protein to mRNA LNP immunization, the animals that received soluble protein in adjuvant showed slightly higher and accelerated serum antibody binding responses compared to their HIV-strain-matched mRNA groups (**Figure 5B**). Serum titers of the animals inoculated via mRNA LNPs did not reach a saturation point even after 4 inoculations, suggesting that perhaps they may have benefited from additional inoculations or a higher dose (50) (**Figure 5A**).

**Figure 6:**
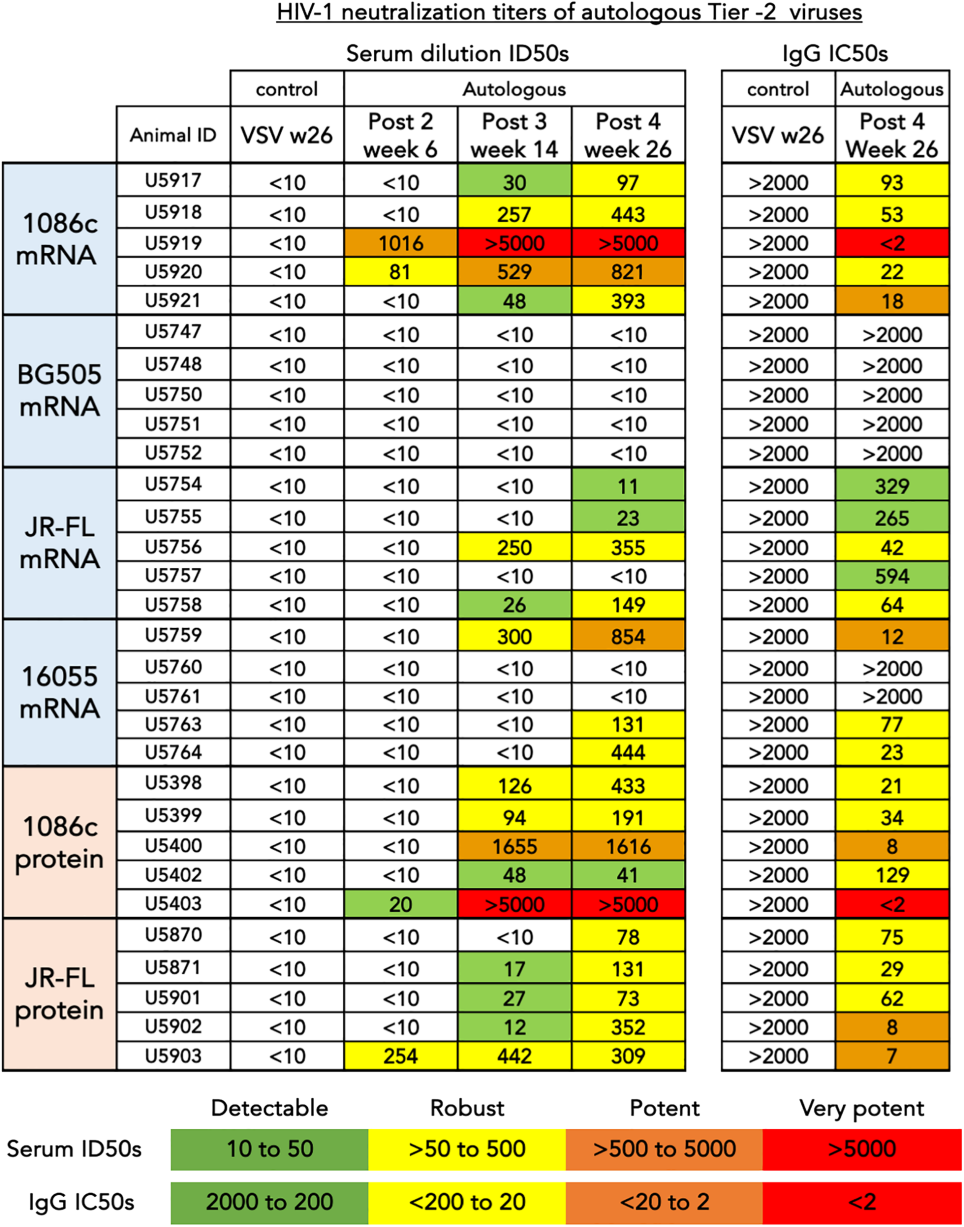
HIV-1 neutralization titers of autologous tier-2 virus. (left) Serum dilution ID50s are shown for three consecutive time points corresponding to bleeds collected after the second, third and fourth inoculations. (Right) Purified total IgG inhibitory concentrations, IC50s are shown after the fourth inoculation for comparison and confirmation of inhibition of entry mediated by immunoglobulin. The values shown represent the serum dilution (left) or the concentration (µg/mL) of total IgG (right) necessary to inhibit 50% of viral entry in a HIV-1 pseudovirus TZM-bL assay. An arbitrary colorimetric scale was used to highlight the variation in neutralization potency of both the serum and IgG samples.

Additionally, we tested the serum for inhibition of HIV entry in a TZM-bl neutralization pseudovirus assay (see Methods). This assay was performed in two different ways: with serum samples (post 2 week 6, post 3 week 14, and post 4 week 26) serially diluted from a starting dilution of 1:10 and then confirmed with protein-A-purified serum IgG (post 4 week 26) starting at 2000 µg/mL of total IgG (**Figure 6**). The total IgG starting dilution is based in the average content of IgG in serum, approximately 10–15 mg/mL. This starting IgG concentration (2000 µg/mL) is approximately equivalent to a 1:5 serum dilution in its capacity to inhibit HIV entry, so about a 2-fold gain in potency and detectability with respect to our serum assays. A low dilution of the sera is equivalent in potency to a higher concentration of the purified total IgG, essentially the ID50s and IC50s are inverted in value (**Figure 6**). Taking both neutralization formats into account, autologous tier-2 virus neutralization was detected in three out of four groups of animals receiving mRNA except for the low binding titer BG505 group of animals (**Figure 6**). The autologous neutralization activity was detected after 2 or 3 inoculations in selected animals and improved with subsequent immunizations (**Figure 6**, left). The strongest neutralizing titers were observed in the 1086c group where all five animals generated strong autologous tier-2 neutralizing titers (**Figure 6**). Likewise, all five animals immunized with JR-FL NFL.711 mRNA generated autologous tier-2 neutralization antibody responses that varied in magnitude as detected in the purified IgG assay format (**Figure 6**, right). Three out of five animals immunized with 16055 NFL.711 mRNA generated robust autologous responses in agreement with past observations in rabbits utilizing stabilized 16055 NFL.664 soluble trimers as immunogens (**Figure 6**) (35). We did not observe a correlation between binding titers, serum or IgG, and neutralization (**Supplementary Figure S5** in **Supplementary Material**).

When comparing autologous neutralizing antibody titers between groups of animals that received mRNA LNPs to those that received strain-matched adjuvanted soluble protein immunizations, the number of responder animals developing autologous tier-2 1086c and JR-FL neutralization titers was the same after 4 immunizations (**Figure 6**). However, the animals that received JR-FL soluble-protein generated slightly stronger autologous neutralization titers and binding titers (**Figures 5**, **6**, respectively). Neutralizing antibody titers against highly sensitive Tier-1 viruses (MN.03, SF162 and HXBc2) were more robust in the soluble protein groups than in the matched mRNA groups (**Supplementary Figure S6** in **Supplementary Material**). These results suggest that the V3 loop in the trimers, main target of these tier-1 neutralizing serum antibodies, may be less exposed in membrane-anchored NFL trimers than in the soluble counterparts (**Supplementary Figure S6** in **Supplementary Material**). These results agree with observations made in a recently published study comparing CH505 HIV Env immunogens delivered via mRNA LNP to soluble SOSIP trimeric proteins (19).

In sum, we conclude that 1086c, JR-FL and 16055 NFL.711 constructs delivered by mRNA LNPS are immunogenic and generate an autologous tier-2 neutralizing antibody response against the matched HIV-1 strain and equivalent to their Env matched soluble protein immunogens. These neutralization data represent strong evidence that our membrane-bound stabilized NFL.711 trimers expressed from mRNA *in vivo* are close mimics of the native spike.

### EMPEM analysis of serum antibody responses reveals sites of vulnerability in 1086c and JR-FL HIV-1 strains

**Figure 7:**
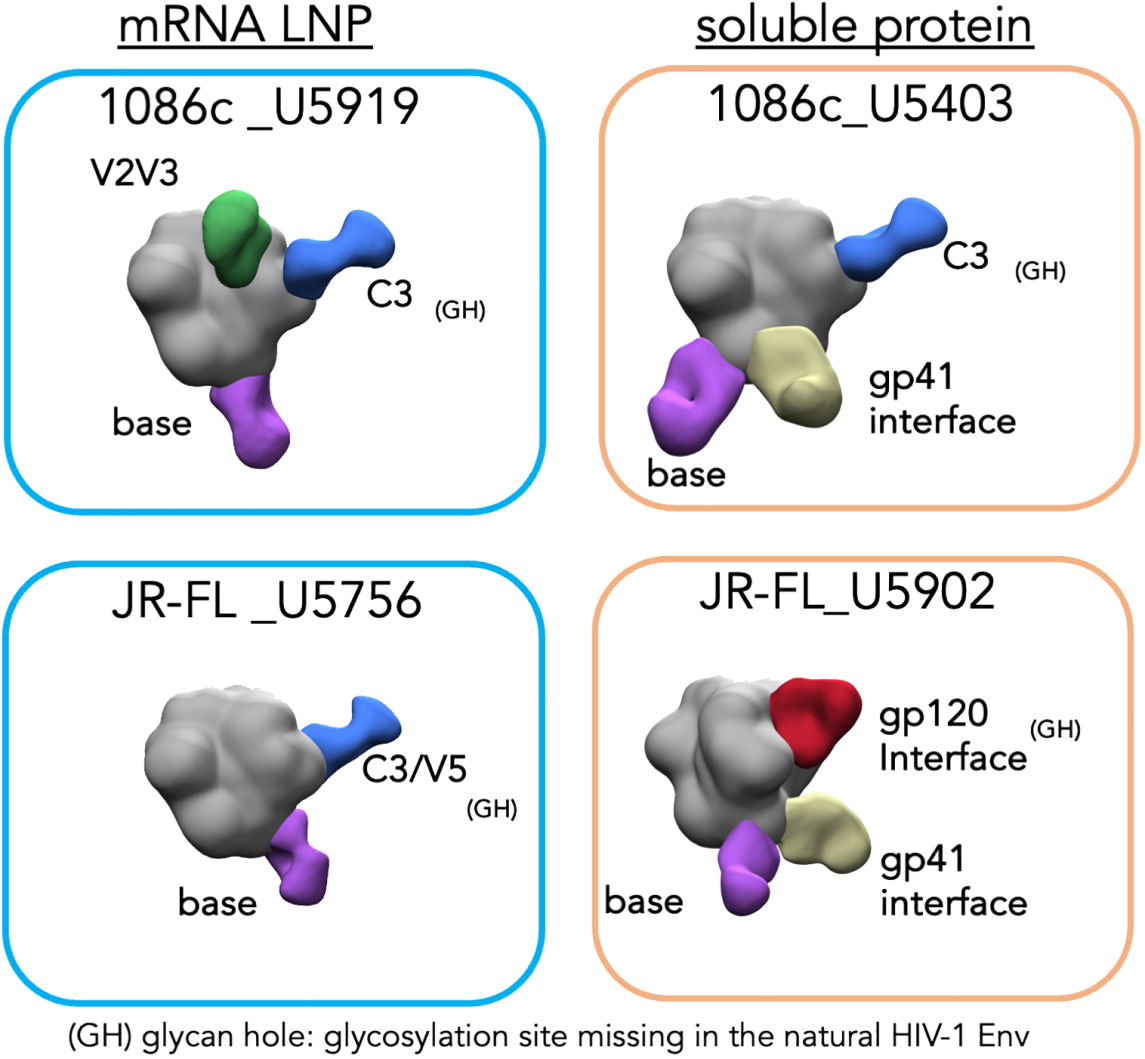
Electron microscopy polyclonal epitope mapping (EMPEM) of selected animal sera. Blue rectangles (left) show the EM densities of the corresponding NFL trimer in complex with the polyFabs from the two animals immunized with mRNA LNPs while orange rectangles (right) show the densities of the animals immunized with the Env (top 1086c and bottom JR-FL) matched soluble proteins. The trimer densities are colored in gray while the polyFabs are colored green, blue, yellow, and purple.

To understand in more detail the vaccine-elicited serum antibody responses by both mRNA and soluble protein immunogens, we selected four animals displaying robust and comparable autologous neutralizing antibody titers representing the two classes of immunogens, mRNA LNPs and soluble protein (animal ID numbers: U5919 and U5403 from the 1086c-based immunizations; and U5756 and U5902 from the JR-FL; respectively, see **Figure 6**) for electron microscopy polyclonal epitope mapping (EMPEM) analysis (51, 52). EMPEM allows us to identify Env epitopes targeted in the vaccine-elicited immune response circulating in the serum IgG. For this analysis, polyclonal IgG was digested to Fabs (polyFab) from the serum samples collected after four immunizations (Post 4 week 26) and combined with the immunogen matched soluble trimer, either 1086c NFL.664 or JR-FL NFL.664. EMPEM revealed several specificities targeting both the 1086c and JR-FL envelope glycoproteins as detected by the polyFab densities bound to trimeric Env (**Figure 7**). For the 1086c Env, polyFab generated by mRNA LNP inoculation displayed three binding targets, two in the gp120 domain (V2V3 region and C3 regions) and a gp41 trimer-base-directed response (**Figure 7**, top left). In comparison, the polyFabs generated in the rabbit immunized with soluble 1086c trimeric proteins displayed the same C3 and trimer base specificities but, in addition, a distinct gp41 interface specificity (**Figure 7**, top right). The C3-directed polyfab density present in both animals overlaps with a natural absence of glycosylation sites at Env positions 356 and 360 in the natural 1086c HIV envelope sequence creating a hole in the glycan shield. Antibodies targeting this glycan hole are likely one of the specificities that mediates the neutralization activity against the 1086c pseudovirus since it shows in the EMPEM analysis as a polyFab (blue) in the samples of the two animals with highest neutralizing antibody titers (**Figures 6**, **7**). Glycan holes resulting from N-glycans absent at specific sites are the main neutralization determinants for other strains of HIV (53, 54). We noted that the mRNA sample showed a V2V3 targeting antibody response, suggesting that membrane-bound trimer array may be beneficial to generate Env-apex targeting serum antibodies as previously demonstrated in a knock-in mouse model (55).

For the JR-FL Env, the mRNA samples revealed two specificities being targeted, the C3/V5 region in gp120 and a base-directed gp41 response (**Figure 7**, bottom left). In contrast, the soluble protein sample showed three specificities, a gp120 interface, a gp41 interface and a trimer base-directed response. The C3/V5 region of gp120, located adjacent to the CD4bs has been identified as a neutralization determinant for other strains of HIV (56). In JR-FL Env this region is missing a glycosylation site at N460 which might explain the elicitation of antibodies targeting the V5. Similarly, the gp120 interface in the JR-FL Env is missing a glycosylation site at position 197 creating a glycan hole that may allow access to a generally well-occluded epitope. The only polyFab density found in both samples was to the trimer-base which generally corresponds to not neutralizing or weakly neutralizing antibody responses. More likely, the EM densities of PolyFabs targeting the C3/V5 and the gp120 interface correspond to antibodies mediating autologous neutralization of HIV-1 JR-FL. Both Env sites lack N-glycans at residues N460 and N197, respectively.

**Figure 8:**
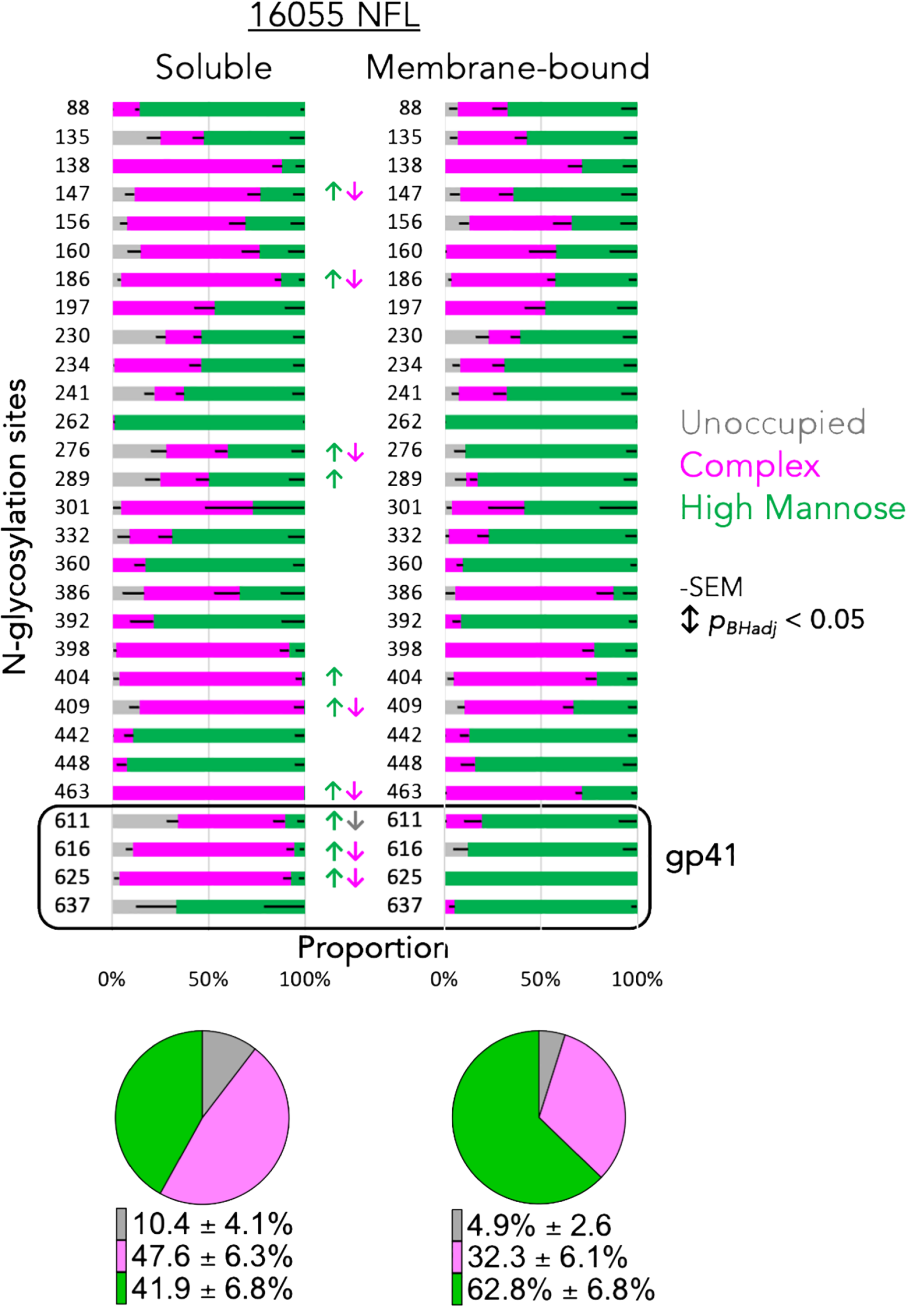
Glycan profiles of the soluble and membrane-bound NFL trimers. Each horizontal bar displays the proportion of unoccupied (gray), complex (magenta) or high-mannose (green) N-glycans at the Env position numbered to the left of the bar. Significant changes in glycan content are indicated by color coordinated arrows. Pie charts display the average content of unoccupied, complex and high-mannose percentage detected in the two variants of the trimeric NFL proteins, soluble (left) and membrane-bound (right).

To note, the EMPEM data derived from the animals that were immunized with soluble protein showed unique densities targeting the gp41 interface (yellow polyFab density, **Figure 7**, right), which are absent from the samples derived from animals that received mRNA LNP vaccinations (**Figure 7**, left). This gp41 density is associated with a non-neutralizing antibody response towards a gp41 region generally occluded by N-glycans at residues 611, 616 and 637. It is viewed as an undesirable consequence of vaccination with soluble Env proteins displaying gp41 N-glycan sites not fully occupied (56–58). Regarding this, we analyzed the glycan profiles of the soluble and membrane-bound 16055 NFL trimers by mass spectrometry (28). This analysis revealed significant differences in the composition and occupancy of N-glycosylation sites between the soluble and membrane-bound NFL trimers (**Figure 8**). We observed statistically significant increases in N-glycan occupancy at the 611 N-glycan site in the membrane-bound trimer compared to the soluble trimer that might explain the absence of the gp41 targeting polyFabs in the samples derived from the mRNA vaccinations. Moreover, the gp41 N-glycan sites presented significant increases in the proportion of high-mannose N-glycans and decreases in complex N-glycans in the membrane-bound trimer suggesting that the anchorage in the membrane or the rigidity of the quaternary structure of these stabilized trimers might sterically hinder access of glycan processing enzymes to the base of the trimer. The high-density of oligomannose glycans in the membrane-bound trimer is consistent with a native-like trimer conformation where glycan processing enzymes have limited accessibility as it has been previously shown (59) (**Figure 8**).

In conclusion, EMPEM analysis revealed several distinct Env sites targeted by serum antibodies of immunized animals whose sera exhibited potent autologous virus neutralization. The polyFab densities mostly targeted sites lacking N-glycans in its respective HIV-1 strain Env (**Figure 7**). As others have reported for other HIV strains (53, 54), these data suggest that naturally lacking N-glycan sites are likely the main sites of vulnerability for the HIV strains studied here, 1086c and JR-FL.

## Discussion

This study represents a natural progression on HIV-1 Env design given the success of mRNA vaccines to prevent severe COVID by limiting SARS-2 replication. The advances in HIV Env vaccine design presented here allow us to deliver trimeric Env immunogens by genetic means in the form of mRNA lipid nanoparticles. These designs build on our soluble, cleavage independent Env trimeric platform NFL. As mentioned, this is an important advantage in the context of genetic vaccines as interventions on purification of relevant Env forms is not possible. For this, we employ a stabilized form of the NFL trimer design, which we successfully used in the past to make well-ordered native-like soluble trimers, linking this construct to the natural HIV-1 transmembrane via a small second linker or, alternatively, restoring the membrane proximal external region to complete the gp41 ectodomain. Note, that older non-stabilized NFL designs generate heterogenous oligomers that do not exclusively retain native-like conformations (9, 21). Homogeneity and fidelity to the HIV native Env conformation are key elements for the success of a genetically delivered HIV vaccine. Here, we utilize our most advanced cassette of trimer stabilization design which aims at increasing trimer stability and homogeneity without altering natural antigenic surfaces (10). We generated four NFL.711 cell surface Env constructs from four HIV-1 strains 1086c, BG505, 16055 and JR-FL and created lipid nanoparticles to test the capacity of these new Env mimics to generate a HIV-1 neutralizing antibody response. While the 16055, 1086c and JR-FL NFL.711 membrane-bound trimers delivered as mRNA LNPs generated an autologous neutralizing antibody response, the construct BG505 NFL.711 did not. We can only speculate that the weak immunogenicity response from the BG505 NFL construct delivered as mRNA LNPs was due to the limited expression of that Env on the cell surface as we demonstrated that most of that Env is retained inside the cell. Our BG505 NFL.711 construct did express adequately from DNA but not from mRNA LNPs. Although the amino acid sequence of these two constructs were identical, the nucleic acid sequence was not, and it is possible that the sequence optimization for mRNA expression reduced translation, translocation to the ER or folding. Indeed, a recent publication demonstrates that modified ribonucleotides can affect fidelity of mRNA translation (60).

Both matched mRNA and soluble protein immunogens (1086c and JR-FL Env-derived constructs) generated very similar autologous neutralizing antibody responses but, as observed before and by others, each individual animal responded with different magnitude titers in both modalities of the vaccine. The difficulty in penetrating the HIV Env glycan shield might account for the variation between animal antibody responses. Our EMPEM analysis, although limited to four animals, suggested that glycan holes are Env regions frequently targeted by B cells in the two HIV strains studied here, 1086c and JR-FL. These results agree with others who have shown similar targets for other HIV strains (53, 54). The EMPEM analysis of the samples derived from soluble protein immunogens but not the mRNA samples did show an additional target in the gp41 interface region (denoted by the yellow density in the right panels of **Figure 7**) where several glycosylation sites are present suggesting that perhaps the membrane bound Env is glycosylated differently in that region, or that the cell membrane on which the trimers are anchored presents a physical barrier to the elicitation of these gp41-directed antibodies. Our glycan profile analysis seems to support the former explanation as glycan occupancy and composition were especially different in the trimer base gp41 region. Another interesting observation is that the antibodies targeting the base of Env were present in the mRNA samples, which suggests that at some point, either when the NFL.711 expressing cell is dying or perhaps when trimers are eventually disassembled by proteases the base epitopes are exposed. However, visual inspection of the 2D class averages in the EMPEM analysis revealed that the mRNA LNPs generated less polyfab densities targeting the trimer base than the soluble trimer samples.

The NFL.711 cell surface expressed HIV-1 trimers studied here represent another important tool to generate an HIV-1 vaccine. Genetic expression of HIV Env can perhaps facilitate the process of generation of clinical-grade reagents to use in heterologous prime:boosting regimens that will likely be necessary to generate an efficacious HIV vaccine.

## Data availability statement

The EMPEM data presented in the study are deposited in the Electron Microscopy Data Bank (EMDB) under accession codes EMD-42516, EMD-42517, EMD-42518 and EMD-42519. The Proteomics data have been submitted to the UCSD MassIVE repository (MSV000094915) with the proteomeXchange ID number PXD052769.

## Ethics statement

The animal study was approved by IACUC LABCORP, 465 Swamp Bridge Rd, Denver, PA 17517. The study was conducted in accordance with the local legislation and institutional requirements.

## Author contributions

JG: Conceptualization, Data curation, Formal analysis, Investigation, Project administration, Supervision, Visualization, Writing – original draft, Writing – review & editing, Methodology. MA: Investigation, Methodology, Writing – review & editing, Conceptualization, Data curation, Formal analysis, Visualization. YF: Investigation, Writing – review & editing, Conceptualization. MA: Investigation, Writing – review & editing, Resources, Methodology. WL: Investigation, Writing – review & editing, Methodology, Resources. SB: Investigation, Writing – review & editing, Formal analysis, Resources. JC: Writing – review & editing, Investigation. RW: Investigation, Writing – review & editing. SB: Resources, Writing – review & editing. GO: Resources, Writing – review & editing, Investigation. PL: Resources, Writing – review & editing. YT: Resources, Writing – review & editing. JD: Methodology, Writing – review & editing. JY: Supervision, Writing – review & editing. JP: Supervision, Writing – review & editing. AW: Methodology, Resources, Supervision, Writing – review & editing. DW: Investigation, Methodology, Resources, Supervision, Writing – review & editing. RW: Conceptualization, Funding acquisition, Resources, Supervision, Writing – original draft, Writing – review & editing.
