# Supplementary material for: mRNA lipid nanoparticles expressing cell-surface cleavage independent HIV Env trimers elicit autologous tier-2 neutralizing antibodies
Source: Front Immunol. 2024 Jul 25;15:1426232. doi: 10.3389/fimmu.2024.1426232 (PMC11306127; doi:10.3389/fimmu.2024.1426232)
Supplement: Supplementary file 1 [file DataSheet_1.docx]

Supplementary Material

## Supplementary Figure 1

**Supplementary Figure 1.** **NFL trimer stabilizing mutations (TD CC+).** All four HIV Env sequences utilized here to make either membrane-bound or soluble NFL proteins were modified as shown in the figure. Each mutation is categorized as followed: TD mutation refers to BG505 Trimer Derived mutation (Guenaga et al, J Virol 2015); V3 refers to V3 loop stabilizing mutations (Guenaga et al, Immunity 2017); FP refers to Fusion Peptide stabilizing mutation (Guenaga et al, Immunity 2017); Helix disrupting refers to proline or glycine residues in gp41 (Sanders et al, J Virol 2002, Guenaga et al, Immunity 2017); Disulfide refers to an introduce Cys-Cys covalent linkage introduced to prevent CD4 conformational changes (Kwon et al, Nat Struct Mol Biol, 2015, Guenaga et al, J. Virol 2015).

**Supplementary Figure 2**

**
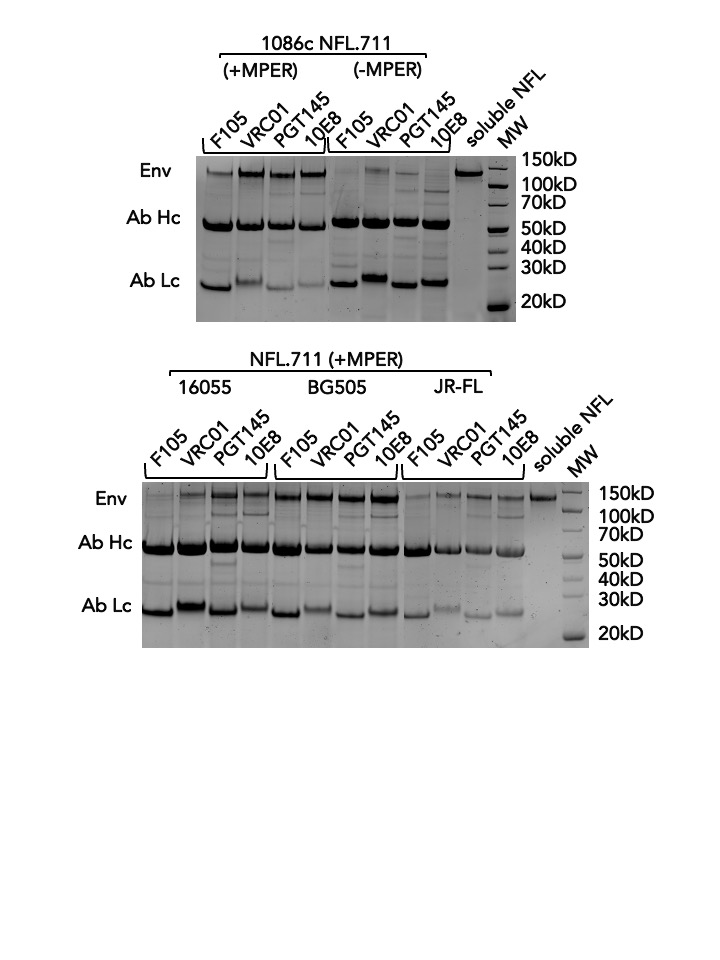
**

**Supplementary Figure 2.** **SDS-PAGE analysis of the immunoprecipitated NFL.711 proteins under reducing conditions.** A panel of 4 antibodies (F105, VRC01, PGT145 and 10E8) were used to selectively purify by immunoprecipitation the membrane bound trimeric Env expressed on the cell surface of HEK 293T cells transfected with the NFL.711 constructs after solubilization with detergent Triton X-100. The different intensity of the Env bands alludes to the selective expression levels of each construct and the affinities of that specific Env for the antibodies tested. Top gel shows a direct comparison of the same 1086c NFL with and without the MPER region. The bottom gel shows the other Env derived NFL.711 (+MPER) 16055, BG505 and JR-FL.

**Supplementary Figure 3**

**
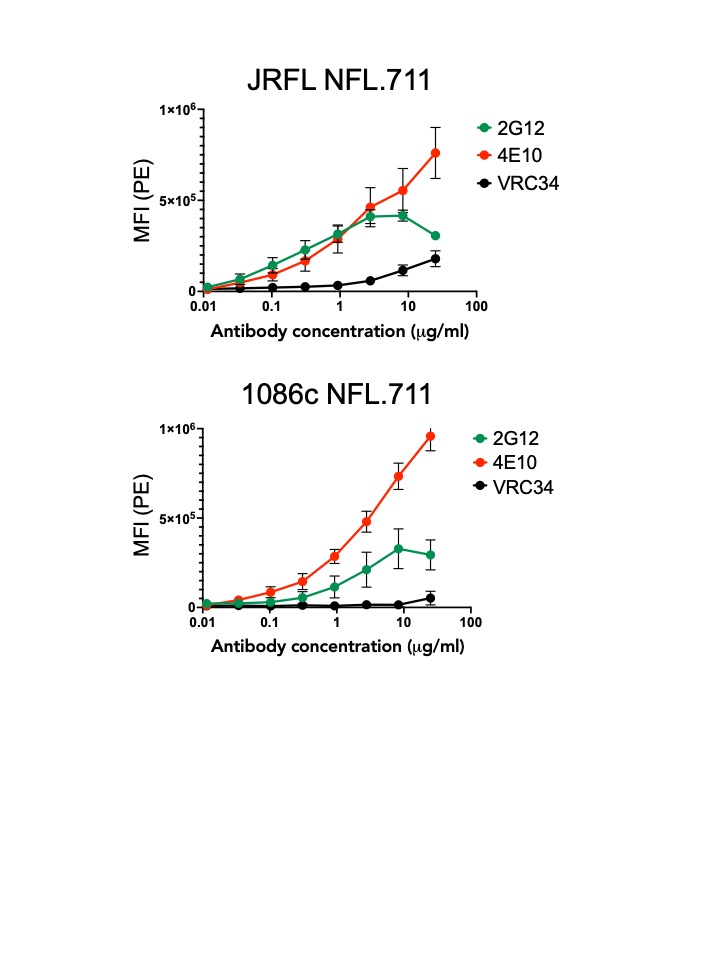
**

**Supplementary Figure 3. FACS binding of selected antibodies to NFL.711 membrane-bound proteins.** The V3-N332 glycan targeting antibody 2G12, the MPER targeting 4E10 and the Fusion Peptide targeting VRC34 were used to assessed binding to JR-FL and 1086c NFLs expressed on the cell surface after DNA plasmid transfection of HEK293 T cells.

**Supplementary Figure 4**

**Supplementary Figure 4.** **Dose dependent expression of the BG505 NFL.711 expressed from mRNA LNPs**. (A) mRNA dose effect on cell-surface expression of the BG505 NFL.711 proteins in transfected HEK 293T cells as assessed by antibodies 10E8 (blue) and F105 (black) with a secondary only negative control. (B) Comparison of cell surface and intracellular expression of BG505 NFL proteins after transfection of HEK293T cells with increasing amounts of mRNA. (C) Relative amounts of cell surface and intracellular proteins after 5μg mRNA transfections of BG505, 16055 and JR-FL measured by mAb binding 10E8.

**Supplementary Figure 5**

**
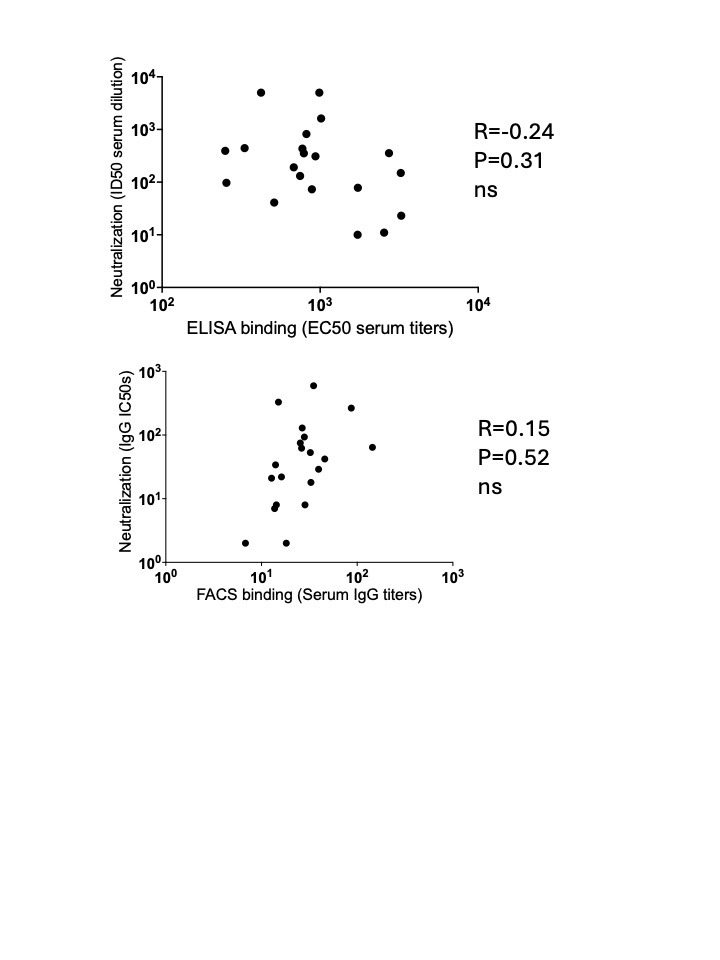
Supplementary Figure 5. Correlation between binding and neutralization.** The top graph displays the non-significant correlation between ELISA serum binding titers and serum neutralization (ID50s) while the bottom graph shows the, also non-significant, correlation between FACS binding of serum IgG titers and the corresponding serum IgG IC50s of neutralization.

**Supplementary Figure 6**

**Supplementary Figure 6.** **Correlation between binding and neutralization.** Animal sera (left) and purified IgG samples (right) were tested in a TZM-bl assay against a panel of tier 1 HIV-1 pseudoviruses (SF162, HXB2 and MN) and VSV as a negative control. Values in the table signify the reciprocal serum dilution (ID50s, left) and purified IgG inhibitory concentrations (IC50s, right) at which 50% of the virus entry is inhibited. An arbitrary colorimetric scale was used to highlight the range of serum and IgG potency of inhibition of viral entry.
